# Non-Markovian Quantum Dynamics in a Squeezed Reservoir

**DOI:** 10.3390/e24030352

**Published:** 2022-02-28

**Authors:** Valentin Link, Walter T. Strunz, Kimmo Luoma

**Affiliations:** 1 Institut für Theoretische Physik, Technische Universität Dresden, D-01062 Dresden, Germany; valentin.link@tu-dresden.de (V.L.); walter.strunz@tu-dresden.de (W.T.S.); 2 Laboratory of Quantum Optics, Department of Physics and Astronomy, University of Turku, FI-20014 Turun Yliopisto, Finland

**Keywords:** open quantum systems, squeezed states, non-Markovian dynamics

## Abstract

We study non-Markovian dynamics of an open quantum system system interacting with a nonstationary squeezed bosonic reservoir. We derive exact and approximate descriptions for the open system dynamics. Focusing on the spin boson model, we compare exact dynamics with Redfield theory and a quantum optical master equation for both short and long time dynamics and in non-Markovian and Markov regimes. The squeezing of the bath results in asymptotic oscillations in the stationary state, which are captured faithfully by the Redfield master equation in the case of weak coupling. Furthermore, we find that the bath squeezing direction modifies the effective system–environment coupling strength and, thus, the strength of the dissipation.

## 1. Introduction

In 1926 and 1927, two families of quantum states for the quantum harmonic oscillator were proposed by Schrödinger [1] and Kennard [2], respectively. The first family of states included coherent states [3,4,5] and the second family included squeezed states [6]. These can be distinguished by considering the Heisenberg uncertainty principle:

$$\Delta x^{2}\Delta p^{2}\geq \frac{\hbar^{2}}{4},$$

which was discovered also in 1927 [7]. The coherent states saturate this uncertainty principle with equal variances $\Delta x^{2}=\Delta p^{2}=\hbar /2$ in both quadratures. This makes them the closest to points in phase space; therefore, coherent states are often considered the most classical quantum states. In contrast, for a squeezed state, the variances of one of the quadratures can be smaller that ℏ/2. The state is, thus, squeezed along a certain direction in phase space. According to the Heisenberg uncertainty principle, the variance of the other quadrature must then be larger than ℏ/2. Often, squeezed states are regarded as nonclassical states [8,9]. In fact, so-called two mode squeezing generates entanglement between two oscillators [10]. In contrast to coherent states, squeezed states are not stationary states of the harmonic oscillator.

Ensembles of harmonic oscillators are commonly considered as quantum environments of open quantum systems. In most applications, these environments are stationary, meaning that the initial state of the reservoir oscillators is a stationary state, for instance, a thermal state (which becomes a coherent state for zero temperature). In a large thermal bath, the system is expected to reach thermal equilibrium [11]. If instead the bath can be prepared in a squeezed state, one directly violates stationarity, resulting in a a breakdown of equilibration.

This can have a drastic impact on the dissipation induced by the bath. In 1986, it was proposed that a nonstationary reservoir consisting of harmonic oscillators prepared in squeezed states can lead to an inhibition of phase decay of an atom [12]. Only in 2013, this prediction was experimentally verified using superconducting qubits [13].

Previous theoretical works on squeezed reservoirs were based on quantum master equations with constant coefficients [14,15,16,17,18,19]. The validity of these equations requires severe assumptions on the system and bath coupling strength and possibly also a separation of time scales such that a rotating wave approximation can be performed. For this reason, such master equations are unable to capture certain phenomena in an exact manner. For instance, they can predict that the very short time dynamics of an observable is linear in time, whereas the Schrödinger equation actually yields quadratic short time dynamics [20,21].

In this article, we investigate open quantum system dynamics in a non-stationary reservoir consisting of oscillators prepared in two-mode squeezed states (broad band squeezed reservoir) [18]. We derive an exact description of the reduced state dynamics using non-Markovian quantum state diffusion (NMQSD) [22,23,24]. Then, using NMQSD as a starting point, we derive a Hierarchy of Equations of Motion [25] (HEOM) for the density matrix of the open system based on the Hierarchy of stochastic Pure States (HOPS) [26]. For the example of a single two level system, we compare the short and long time dynamics of the numerically exact HEOM theory with a weak coupling perturbation theory, so-called Redfield theory, and the commonly used standard master equation with time independent coefficients. Our main findings are that, in a parameter regime where a quantum optical master equation would work fine for stationary reservoirs, it can not capture accurately short and long time dynamics. Remarkably, if the bath memory time is short the Redfield theory performs extremely well, matching with exact dynamics and predicting accurate long time dynamics.

## 2. Model

We consider the following commonly used model for open quantum systems:

(1) $$H=H_{S}+L\sum_{\lambda }g_{\lambda }\left(a_{\lambda }+a_{\lambda }^{†}\right)+\sum_{\lambda }\omega_{\lambda }a_{\lambda }^{†}a_{\lambda },$$

where the environment consists of independent harmonic oscillators (bosonic modes) $\left[a_{\lambda },a_{\mu }^{†}\right]=\delta_{\lambda \mu }$ [19]. The system Hamiltonian $H_{S}$ and the system coupling operator are left arbitrary, except that we demand $L=L^{†}$. Moving to an interaction representation with respect to the free bath Hamiltonian, one obtains the following.

(2) $$\begin{array}{cc} \; & H\left(t\right)=H_{S}+LB\left(t\right),\;B\left(t\right)=\sum_{\lambda }g_{\lambda }\left(e^{-i\omega_{\lambda }t}a_{\lambda }+e^{i\omega_{\lambda }t}a_{\lambda }^{†}\right) \end{array}$$

If the bath’s initial state is Gaussian and the coupling of the system to the bath is linear, the response of the bath to the system is fully characterized by the first and second moment of the bath response operator B(t). Typically, and without loss of generality, one considers 〈B(t)〉=0. Then, the so-called bath correlation function (BCF) is the only relevant quantity describing the influence of the bath [27]:

(3) α(t,s)=〈B(t)B(s)〉,

where the expectation value is with respect to the bath initial state. As an initial condition, for the bath, usually a stationary state of the free bath Hamiltonian is considered. For example, a zero temperature bath is described by vacuum state |0〉, which is a Gaussian state; hence, it is fully characterized by the following correlations.

(4) $$\begin{array}{cc} \; & \langle 0|a_{\lambda }|0\rangle =0,\\ \; & \langle 0|a_{\lambda }a_{\mu }^{†}|0\rangle =\delta_{\lambda \mu },\\ \; & \langle 0|a_{\lambda }^{†}a_{\mu }|0\rangle =0,\\ \; & \langle 0|a_{\lambda }a_{\mu }|0\rangle =0 \end{array}$$

This results in a stationary BCF that depends on the time difference only.

(5) $$\alpha \left(t,s\right)\equiv \alpha_{0}\left(t-s\right)=\sum_{\lambda }g_{\lambda }^{2}e^{-i\omega_{\lambda }\left(t-s\right)}$$

An alternative characterization of a stationary bath is given in terms of spectral density:

(6) $$J\left(\omega \right)=\frac{1}{\pi }\sum_{\lambda }g_{\lambda }^{2}\delta \left(\omega -\omega_{\lambda }\right),$$

which is the Fourier transform of the stationary BCF.

In this article, we are interested in the situation where the initial state of the bath is a squeezed vacuum. In particular, we consider two-mode squeezing which is symmetric around a reference frequency $\omega_{0}$. We denote our squeezed vacuum state by |ϕ〉=S|0〉, where *S* is a unitary squeezing operator. If we order the bath modes according to $\omega_{2\lambda_{0}-\lambda }=2\omega_{0}-\omega_{\lambda }$, the state |ϕ〉 is characterized by the following correlations.

(7) $$\begin{array}{cc} \; & \langle \phi |a_{\lambda }|\phi \rangle =0,\\ \; & \langle \phi |a_{\lambda }a_{\mu }^{†}|\phi \rangle =u^{2}\;\delta_{\lambda \mu },\\ \; & \langle \phi |a_{\lambda }^{†}a_{\mu }|\phi \rangle ={\left|v\right|}^{2}\;\delta_{\lambda \mu },\\ \; & \langle \phi |a_{\lambda }a_{\mu }|\phi \rangle =-vu\;\delta_{\mu ,2\lambda_{0}-\lambda } \end{array}$$

Because *S* is a unitary operator, the squeezing parameters u∈R and v∈C satisfy $u^{2}-{\left|v\right|}^{2}=1$. We also have assumed that the two mode squeezing is homogeneous, i.e., *u* and *v* are the same for all modes λ. This is often called broadband squeezing. In order to parametrize the squeezing, we introduce real variables *r* and φ and write

(8) $$u=\cosh\left(r\right),\;v=\cosh\left(r\right)e^{i\varphi },$$

where *r* is squeezing strength, and ϕ is the squeezing direction in phase space. ϕ=0 corresponds to squeezing the *p*-quadrature, whereas ϕ=π corresponds to squeezing the *x*-quadrature. In case of no squeezing r=0, we recover the vacuum correlations (4). To continue, we further assume that coupling constants $g_{\lambda }$ have a symmetry property $g_{2\lambda_{0}-\lambda }=g_{\lambda }$, which implies that the spectral density is symmetric around $\omega_{0}$. From this, we obtain that the bath correlation function has the following structure:

(9) $$\begin{array}{cc} \alpha (t,s)= & \alpha_{0}\left(t-s\right)\left(u^{2}-vue^{-2i\omega_{0}s}-v^{*}ue^{2i\omega_{0}t}\right)+\alpha_{0}^{*}\left(t-s\right){\left|v\right|}^{2}, \end{array}$$

where $\alpha_{0}\left(t-s\right)$ is the zero temperature bath correlation function from Equation (5). It is easy to check that this is a valid BCF obeying $\alpha \left(t,s\right)=\alpha^{*}\left(s,t\right)$. Note that this function does depend explicitly on both *t* and *s* and not only on their difference. This is because the bath initial state is not stationary with respect to the bath Hamiltonian. We assume the following model for the vacuum BCF:

(10) $$\begin{array}{c} \alpha_{0}\left(t-s\right)=\frac{\gamma \Gamma }{2}e^{-\Gamma |t-s|-i\omega_{B}\left(t-s\right)}, \end{array}$$

which corresponds to a continuous bath with Lorentzian spectral density.

(11) $$J\left(\omega \right)=\frac{\gamma }{2}\frac{\Gamma^{2}}{\Gamma^{2}+{\left(\omega_{B}-\omega \right)}^{2}}$$

This bath satisfies the required symmetry if $\omega_{B}=\omega_{0}$, which we assume in the following. Notably, for such a bath, the white noise limit $\alpha_{0}\left(t-s\right)\to \gamma \delta \left(t-s\right)$ exits when Γ→∞.

The non-Markovian open system dynamics of this model can be described with non-Markovian quantum state diffusion (NMQSD) [22,24,28]. NMQSD is a stochastic unraveling of reduced open system dynamics in terms of a Gaussian colored noise process z(t) with statistics $E\left[z\left(t\right)z^{*}\left(s\right)\right]=\alpha \left(t,s\right)$ and $E\left[z(t)\right]=E\left[z(t)z(s)\right]=0$. The system state is obtained as the ensemble average of stochastic pure states $|\psi (z^{*},t)\rangle$, which depend on this noise process.

(12) $$\rho \left(t\right)=E\left[|\psi \left(z^{*},t\right)\rangle \;\langle \psi \left(z^{*},t\right)|\right]$$

The stochastic states obey the NMQSD evolution equation.

(13) $$\begin{array}{cc} \partial_{t}|\psi \left(z^{*},t\right)\rangle = & \left(-iH_{S}+Lz^{*}\left(t\right)\right)|\psi \left(z^{*},t\right)\rangle -L\int_{0}^{t}ds\;\alpha \left(t,s\right)\frac{\delta }{\delta z^{*}\left(s\right)}|\psi \left(z^{*},t\right)\rangle \end{array}$$

Different solution strategies for this equation exits, notably the *O*-operator method [23,29] and the exact HOPS method [26,30,31]. Here, we use NMQSD as a tool to derive perturbative and exact master equations for the squeezed bath problem (9).

Although most results are very general, an example we will consider is the spin boson model. For this model, the system is a simple two level system with the following operators

(14) $$H_{S}=\frac{\Omega }{2}\sigma_{z},\;L=\sigma_{x}.$$

## 3. Perturbative Master Equations

If the coupling to the bath is weak and/or the BCF decays rapidly, we can make a perturbative approximation to NMQSD. The lowest order perturbative equation is given by the following.

(15) $$\begin{array}{cc} \; & \int_{0}^{t}ds\;\alpha \left(t,s\right)\frac{\delta }{\delta z^{*}\left(s\right)}|\psi \left(z^{*},t\right)\rangle =\bar{O}\left(z^{*},t\right)|\psi \left(z^{*},t\right)\rangle ,\\ \; & \bar{O}\left(z^{*},t\right)\approx \int_{0}^{t}ds\;\alpha \left(t,s\right)e^{-iH_{S}\left(t-s\right)}Le^{iH_{S}\left(t-s\right)}. \end{array}$$

Because in this approximation the operator $\bar{O}$ does not depend on the stochastic process, a master equation can be directly derived from NMQSD as follows [23].

(16) $$\begin{array}{cc} \partial_{t}\rho \left(t\right) & =-i\left[H_{S},\rho \left(t\right)\right]+\left[L,\rho \left(t\right){\bar{O}}^{†}\left(t\right)\right]+\left[\bar{O}\left(t\right)\rho \left(t\right),L\right]\\ \; & :=L_{t}^{R}\left(\rho \left(t\right)\right) \end{array}$$

This is the well known Redfield master equation [32].

Under certain conditions and in a frame rotating with the frequency $\omega_{0}$, one can derive a master equation with constant coefficients starting from the Redfield equation. The resulting equation is well known from quantum optics textbooks [17,18]. For a derivation, we assume the spin boson model with $L=\sigma_{x}$ and $H_{S}=\omega \sigma_{z}/2$. In particular, we consider the case where $\Omega =\omega_{0}+\delta$ and $\omega_{0}$ defines the fastest timescale $\omega_{0}\gg \Gamma \gg \delta$ and $\omega_{0}\gg \gamma$. The state in the rotating frame is given by the following.

(17) $$\tilde{\rho }\left(t\right)=R\left(t\right)\rho \left(t\right)R^{†}\left(t\right),\;R\left(t\right)=\exp\left(i\omega_{0}\frac{\sigma_{z}}{2}t\right)$$

To obtain a master equation with constant coefficients, analogous to the standard quantum optical master equation [32], one explicitly computes the integral in (15) and neglects exponentially decaying terms as well as terms of order δ/Γ. Plugging the result into the Redfield Equation (16) in the rotating frame, one can identify counter-rotating terms that oscillate at frequency $2\omega_{0}$. Under the above assumptions, these terms can be neglected and one obtains a master equation with time-independent coefficients.

(18) $$\begin{array}{cc} \partial_{t}\tilde{\rho }\left(t\right)= & -i\frac{\delta }{2}\left[\sigma_{z},\tilde{\rho }\left(t\right)\right]\\ \; & +\gamma u^{2}\left(\sigma_{-}\tilde{\rho }\left(t\right)\sigma_{+}-\frac{1}{2}\left\{\sigma_{+},\sigma_{-},\tilde{\rho }\left(t\right)\right\}\right)\\ \; & +{\gamma |v|}^{2}\left(\sigma_{+}\tilde{\rho }\left(t\right)\sigma_{-}-\frac{1}{2}\left\{\sigma_{-},\sigma_{+},\tilde{\rho }\left(t\right)\right\}\right)\\ \; & +\gamma uv\sigma_{-}\tilde{\rho }\left(t\right)\sigma_{-}+\gamma uv^{*}\sigma_{+}\tilde{\rho }\left(t\right)\sigma_{+}\\ := & L^{M}\left(\tilde{\rho }\left(t\right)\right) \end{array}$$

Note that the coefficients of the last two terms become time dependent if one moves back to the laboratory frame.

In order to assess the validity of these perturbative master equations, we have to compare their predictions with exact results. We discuss in the following how the exact reduced dynamics can be computed using non-Markovian open system methods.

## 4. Exact Method

We can utilize non-Markovian open system methods to compute the exact reduced dynamics in the squeezed reservoir. In particular, due to the exponential form of the bath correlation function, we can easily generalize hierarchical methods for the squeezed bath. To this aim, we decompose the BCF in the following manner:

(19) $$\alpha \left(t,s\right)=\alpha_{1}\left(t,s\right)+\alpha_{2}\left(t,s\right)$$

(20) $$\begin{array}{cc} \; & \alpha_{1}\left(t,s\right)=\frac{\gamma \Gamma }{2}\left(u^{2}-vue^{-2i\omega_{0}s}\right)e^{-i\omega_{0}\left(t-s\right)-\Gamma \left|t-s\right|} \end{array}$$

(21) $$\begin{array}{cc} \; & \alpha_{2}\left(t,s\right)=\frac{\gamma \Gamma }{2}{\left(|v\right|}^{2}-v^{*}ue^{2i\omega_{0}s})e^{i\omega_{0}\left(t-s\right)-\Gamma \left|t-s\right|} \end{array}$$

To solve the linear NMQSD Equation (13), we further define the corresponding functional differential operators:

(22) $$D_{j}=\int_{0}^{t}ds\;\alpha_{j}\left(t,s\right)\frac{\delta }{\delta z_{s}^{*}},\;j=1,2,$$

so that (13) becomes the following.

(23) $$\partial_{t}|\psi \rangle =-iH|\psi \rangle +Lz_{t}^{*}|\psi \rangle -L\sum_{j=1,2}D_{j}|\psi \rangle$$

Because of the exponential form of the kernels (20), we can compute the time derivative of the functional differential operators.

(24) $$\begin{array}{cc} \; & \partial_{t}D_{j}=-W_{j}D_{j}+\alpha_{j}\left(t,t\right)\frac{\delta }{\delta z_{t}^{*}},\;W_{1}=W_{2}^{*}=i\omega_{0}+\Gamma \end{array}$$

This allows employing the hierarchy of pure states (HOPS) scheme to solve NMQSD. In particular, one defines an ’auxiliary state’ for every double-index $n\in N_{0}^{2}$.

(25) $$|\psi^{n}\rangle ={\left(D_{1}\right)}^{n_{1}}{\left(D_{2}\right)}^{n_{2}}|\psi \rangle$$

These states obey the hierarchy of pure states equation of motion.

(26) $$\begin{array}{cc} \partial_{t}|\psi^{n}\rangle = & \left(-iH_{S}+Lz_{t}^{*}-\sum_{j=1,2}n_{j}W_{j}\right)|\psi^{n}\rangle -L\sum_{j=1,2}|\psi^{n+e_{j}}\rangle +L\sum_{j=1,2}\alpha_{j}\left(t,t\right)n_{j}|\psi^{n-e_{j}}\rangle \end{array}$$

In this equation, we set ${\left(e_{j}\right)}_{i}=\delta_{ij}$. The exact NMQSD solution is simply the ’root’ state of the hierarchy $\left|\psi \rangle =\right|\psi^{\left(0\right)}\rangle$. Truncating the hierarchy at a finite depth yields a closed set of equations, which can be numerically integrated. Typically, for a weak coupling, only a few auxiliary states have to be taken into account to achieve convergence. There also exists a hierarchy of operators complementary to HOPS, which is known as the hierarchy of equations of motions (HEOM) [25,30]. We define auxilliary operators with two multiindices (n,m) as follows.

(27) $$\rho^{\left(n,m\right)}=E\left[|\psi^{\left(n\right)}\rangle \;\langle \psi^{\left(m\right)}|\right]$$

Upon employing the HOPS Equation (26) with relation $E\left[z_{t}\ldots \right]=E\left[\sum_{j}D_{j}\ldots \right]$, we find the hierarchical equations of motion:

(28) $$\begin{array}{cc} \partial_{t}\rho^{\left(n,m\right)}= & -i\left[H,\rho^{\left(n,m\right)}\right]-\sum_{j=1,2}\left(n_{j}W_{j}+m_{j}W_{j}^{*}\right)\rho^{\left(n,m\right)}\\ \; & +\sum_{j=1,2}\left(n_{j}\alpha_{j}\left(t,t\right)L\rho^{\left(n-e_{j},m\right)}+m_{j}\alpha_{j}^{*}\left(t,t\right)\rho^{\left(n,m-e_{j}\right)}L\right)\\ \; & +\sum_{j=1,2}\left(\left[\rho^{\left(n+e_{j},m\right)},L\right]+\left[L,\rho^{\left(n,m+e_{j}\right)}\right]\right), \end{array}$$

where the reduced state of the system is simply given by $\rho \left(t\right)=\rho^{\left(0,0\right)}\left(t\right)$. We can use a truncated hierarchy to accurately describe the non-Markovian open system dynamics of the model, even for strong coupling (large γ) and long memory time (small Γ).

## 5. Dynamics

The fidelity $F_{t}=|\langle \psi_{0}\left|\rho \left(t\right)\right|\psi_{0}{\rangle |}^{2}$ of time-evolved state ρ(t) with an initial pure system state $|\psi_{0}\rangle$ can be used to investigate how master equations capture short time dynamics. In general, the fidelity behaves for short times as [20]:

(29) $$\begin{array}{c} F_{t}^{F}=1-\left(\Delta H_{S}^{2}+\Gamma_{F}\right)t^{2}, \end{array}$$

where $\Gamma_{F}=2\alpha \left(0,0\right)$ and $\Delta X^{2}=\langle X^{2}\rangle -{\langle X\rangle }^{2}$ are the variances of operator *X*.

A quantum optical master equation that has constant coefficients, such as Equation (18), instead, leads to a linear short time behavior for the fidelity of the state of the system alone [21]:

(30) $$\begin{array}{c} F_{t}^{M}=1-\Gamma_{M}t+O\left(t^{2}\right), \end{array}$$

where $\Gamma_{M}=\sum_{\mu }c_{\mu }\left(\langle R_{\mu }L_{\mu }\rangle -\langle R_{\mu }\rangle \langle L_{\mu }\rangle \right)$. Here, operators $L_{\mu },\;R_{\mu }$ are the operators, which act on the left or on the right, respectively, in the sandwich term of the master equation.

The quadratic short time behavior is captured exactly by the Redfield theory, which is the lowest order perturbation theory.

(31) $$\begin{array}{c} F_{t}^{R}=1-\left(\Delta H_{S}^{2}+\Gamma_{F}\right)t^{2} \end{array}$$

**Figure 1:**
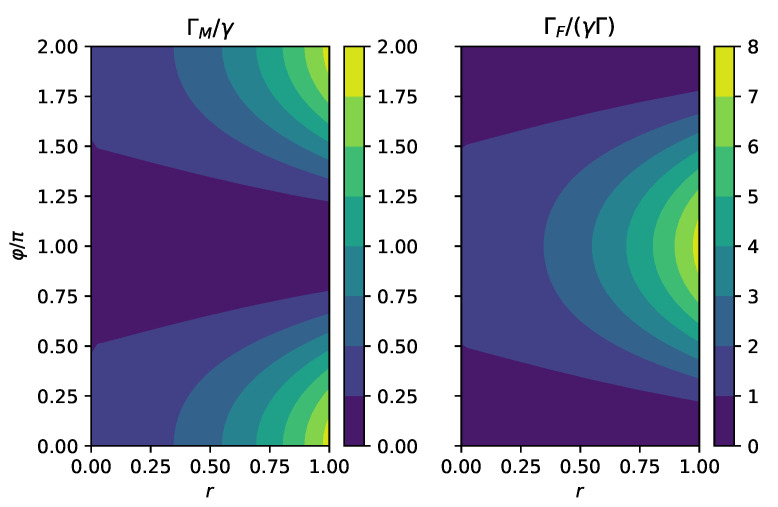
(**Left**): Linear rate $\Gamma_{M}$ for short time dynamics due to the Markov master equation divided by the overall coupling strength γ. (**Right**): Quadratic rate for short time dynamics for the total system $\Gamma_{F}$ divided by γΓ, where Γ is the inverse bath correlation time scale. $\Gamma_{F}$ is proportional to ${\left|u-v\right|}^{2}$, which has a maximum when φ=π. $\Gamma_{M}$ is proportional to $u(v+v^{*})$, which has a minimum at φ=π. Here, we consider the spin boson model (14) in a squeezed bath (9) with u=cosh(r) and $v=\sinh\left(r\right)e^{i\varphi }$ and initial state $|\psi_{0}\rangle =|+\rangle :=\frac{1}{\sqrt{2}}(|0\rangle +\left|1\rangle \right)$, where |0〉 and |1〉 are the eigenstates of $\sigma_{z}$. We have chosen the following parameters: r=1/2, Γ=5γ, and $\Omega =\omega_{B}=\omega_{0}=\gamma$.

In Figure 1, we compare the short time linear rate $\Gamma_{M}$ computed for master Equation (18) and the quadratic rate $\Gamma_{F}$ for the full system. We observe that the full rate has a maximum at φ=π, which corresponds to squeezing along the *x*-direction. The two-level system is coupled to the *x*-quadrature of the bath, and squeezing in this direction increases the coupling strength which should lead to faster decay. In contrast, the master equation predicts a minimum for ϕ=π, which is not in line with the microscopic theory.

**Figure 2:**
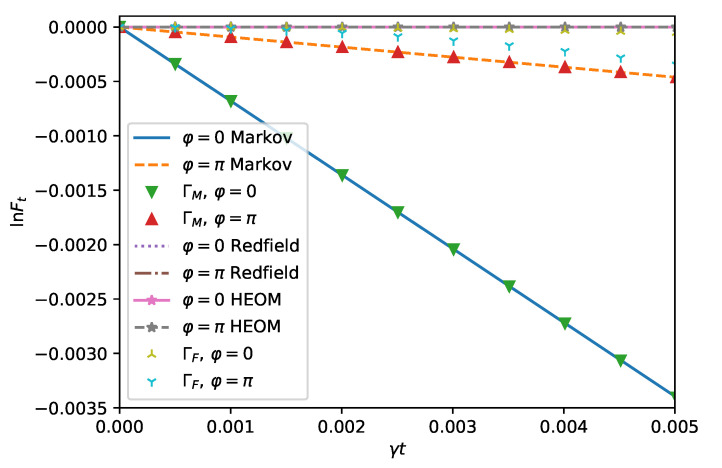
Short time dynamics of the spin boson model (14) in the squeezed bath (9) with Γ=5γ, $\Omega =\omega_{B}=\omega_{0}=\gamma$, r=0.5, and $|\psi_{0}\rangle =|+\rangle$. The logarithm of the fidelity $F_{t}$ is displayed for short times within different approximate and exact descriptions. The quadratic short time dynamics holds up to γt=0.002 for φ=π as the HEOM and the Redfield theory start to deviate from the short time expansion. For φ=0, the quadratic short time dynamics, HEOM, and the Redfield agree well in the range of the plot. This is explained by the effectively strong system environment coupling for φ=π, which sets a different regime of validity for the short time expansion. The parameters are chosen as in Figure 1.

In Figure 2, we compare the logarithm of the fidelity computed using the HEOM (28), the Redfield theory (16), and the master equation (18) with quadratic short time estimates (29), (31) and the linear estimate (30).

The quadratic estimates; the HEOM and the Redfield curves are in line with each other for both squeezing directions φ=0 and φ=π. The linear prediction and the master equation curves also are on top of each other but show a different behavior for different ϕ. For φ=0, the decay of the fidelity is faster than for φ=π, as expected from Figure 1.

**Figure 3:**
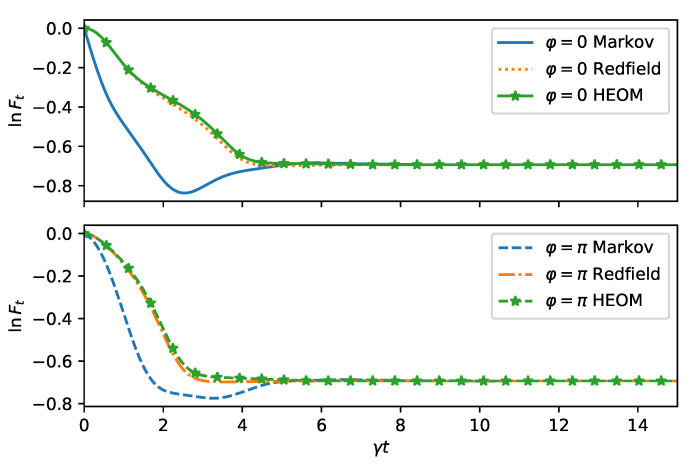
Dynamics of the fidelity as in Figure 2 but for long times and for two squeezing directions φ=0 (**top**) and for φ=π (**bottom**). The decay of the fidelity is faster for φ=π for HEOM and Redfield as expected from the microscopic model. Predictions from the quantum optical master equation (Markov) show an opposite behavior and the fidelity even has a positive slope at intermediate times.

For longer times, the fidelity is still captured correctly by the Redfield equation, while the Markovian equation becomes valid only asymptoically, as seen in Figure 3.

**Figure 4:**
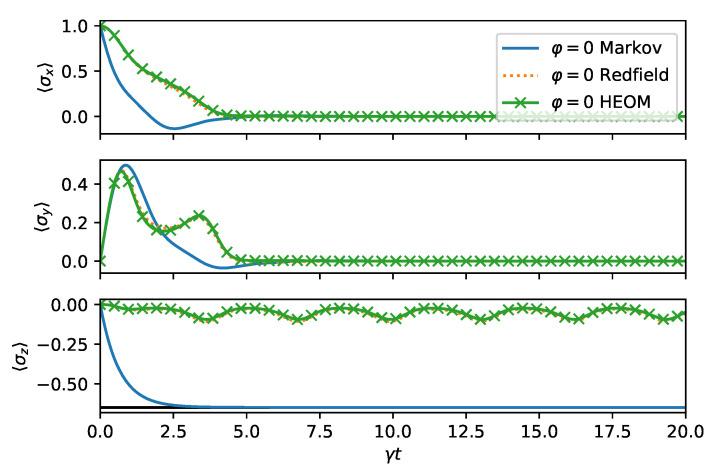
Long time dynamics of the spin boson model (14) in the squeezed bath (9) as in Figure 2 with φ=0. Both approximate methods describe correctly that the *x* and *y* Bloch sphere components decay to zero. The $\sigma_{z}$ expectation value asymptotically acquires a finite value modulated by weak oscillations which are captured properly in the Redfield theory.

**Figure 5:**
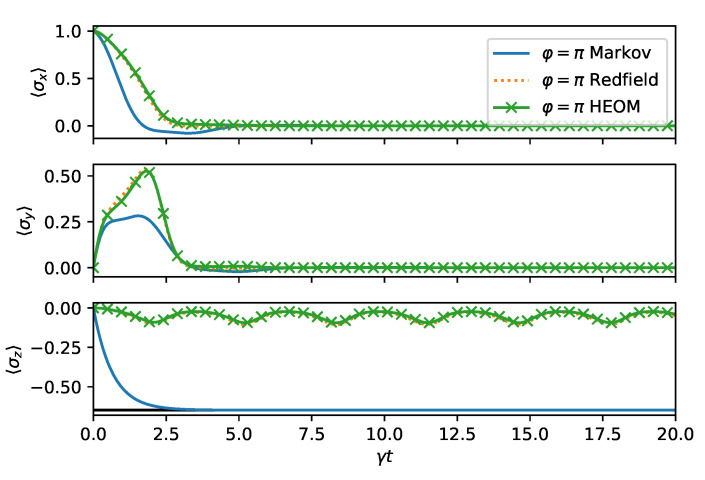
Long time dynamics as in Figure 4, except φ=π.

We now turn to the long time dynamics, which reveals the steady state properties. In Figure 4, we compare the time evolution computed from HEOM, Redfield, and master equation for φ=0. We do the same for φ=π in Figure 5. The same conclusions hold as before: For φ=π, the decay is faster, as can be observed from HEOM and Redfield curves. The steady state prediction of the master equation is far-off from the Refield and HEOM computations, which both agree exptremely well for the choosen parameters. This is because the memory time of the bath 1/Γ is chosen to be very short. As expected for a spin boson model, the steady state reaches a finite $\langle \sigma_{z}\rangle$ value. However, due to the non-stationary bath, some residual oscillations remain indefinitely in long time dynamics.

**Figure 6:**
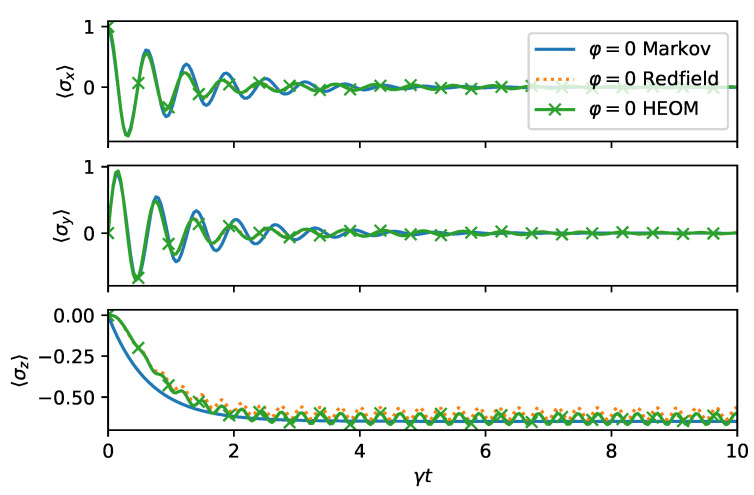
Dynamics of the spin boson model (14) in the squeezed bath (9) with Γ=3γ, Ω=10γ, $\omega_{0}=\Omega$, r=0.5, φ=0, and initial state $|\psi_{0}\rangle =|+\rangle$. The Markovian master Equation (18) agrees well with the exact dynamics but does not capture small asymptotic oscillations.

Next, we investigate the Markov limit where we expect the master Equation (18) to be applicable. We chose $\Omega =\omega_{0}=10\gamma$, Γ=3γ, which is in a regime where the assumptions for the rotating wave approximation are satisfied. Moreover, since Γ/γ=3, the BCF decays faster than the time scale set for system bath interaction by γ. As we can observe from Figure 6, the master equation, the Redfield theory, and HEOM are in good qualitative agreement. The rapid oscillations present in HEOM and Redfield solutions are removed by the secular approximation behind the Markovian master equation.

**Figure 7:**
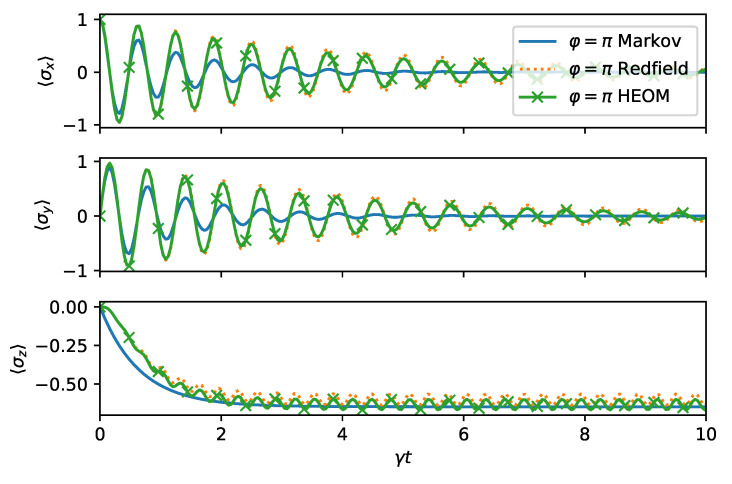
Dynamics of the spin boson model as in Figure 6, except φ=π.

When we change the direction of the squeezing to φ=π, hence effectively coupling the system and the environment more strongly, the agreement is not as good, as shown in Figure 7. This in particularly visible for the expectation value $\langle \sigma_{y}\rangle$. The asymptotic values are, however, captured accurately.

**Figure 8:**
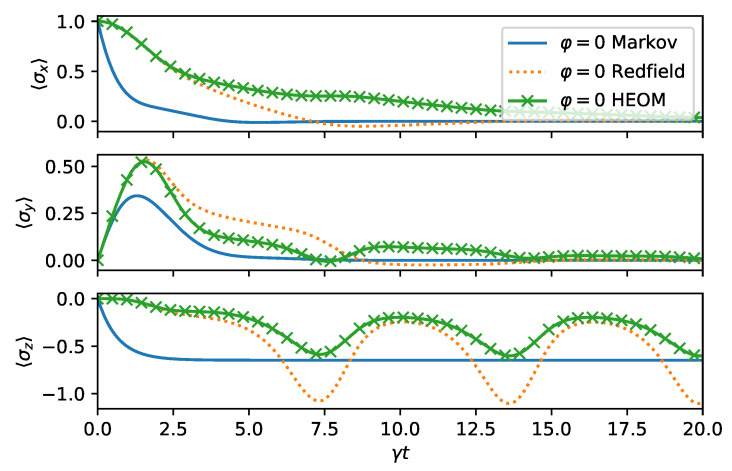
Highly non-Markovian dynamics in the spin boson model. We have chosen $|\psi_{0}\rangle =|+\rangle$, Γ=γ/2, $\Omega =\omega_{B}=\omega_{0}=\gamma /2$, and φ=0. We are in the strong system–envinronment coupling regime since γ>Γ. This means that system–environment dynamics occurs on a faster time scale than the bath correlation function decay time. The failure of weak coupling master equations is expected.

Lastly, we investigate a regime for non-Markovian dynamics. The Redfield theory is a weak system–environment coupling theory. We expect it to fail when the bath correlation function decays on a slower time scale than the system environment interactions; that is, when Γ<γ. For short times, the Redfield theory agrees with the exact results. We confirm these statements in Figure 8, where one observes that the Redfield and the quantum optical master equation are both far-off from the exact solution. In fact, in this regime, the solution of the Redfield equation ceases to be positive, as can be observed from $\langle \sigma_{z}\rangle <-1$. To compute the exact dynamics, we have estimated convergence of HEOM by increasing the hierarchy depth until the relative change in the solution is less than $10^{-2}$.

## 6. Conclusions

In this article, we have revisited an old quantum optical model, namely, the decay to a squeezed reservoir [12]. This model has been realized experimentally using superconducting qubits [13] and is an interesting example of non-stationary open system dynamics. We investigated the short and long time dynamics of the model using textbook master equations [17,18,19], perturbative Redfield theory [32], and exact hierarchical methods [25,26], which we generalize to treat our non-Markovian squeezed bath model. Assuming broadband two mode squeezing, a model for the bath correlation function can be proposed where the bath memory time can be easily controlled by a single parameter Γ. This corresponds to a Lorentzian spectral density, which yields an exponentially decaying bath correlation function. We showed how in the limit where Γ≫γ, where γ is the overall system environment coupling, the Redfield theory performs extremely well. However, the dynamics is far from a regime where a usual “quantum optical master equation” is valid. This is due to the fact that there are other time scales involved which need to satisfy a strict hierarchical order so that the Markov approximation can be justified. These timescales are determined by the free system evolution (Ω) and the bath resonance ($\omega_{0}$). The quantum optical master equation is valid only in a regime where $\omega_{0}$ is by far the fastest time-scale. All in all, the Redfield theory, which is based on weak coupling approximation only, produces the correct short and long time dynamics in a wide parameter range and is conceptually and numerically much simpler then more advanced methods such as HEOM. To those concerned about the possible positivity violations when using the Redfield theory, we would like to point out that this should be seen merely as a symptom of the breakdown of the weak coupling perturbation theory [32].

## Data Availability

Not applicable.

## Abbreviations

The following abbreviations are used in this manuscript:

NMQSD	Non-Markovian quantum state diffusion;
HEOM	Hierarchical equations Of motion;
HOPS	Hierarchy of pure states;
BCF	Bath correlation function.
